# Interaction of natural killer cells with neutrophils exerts a significant antitumor immunity in hematopoietic stem cell transplantation recipients

**DOI:** 10.1002/cam4.550

**Published:** 2015-11-21

**Authors:** Ryosuke Ueda, Kenta Narumi, Hisayoshi Hashimoto, Reina Miyakawa, Takuji Okusaka, Kazunori Aoki

**Affiliations:** ^1^ Division of Molecular and Cellular MedicineNational Cancer Center Research Institute5‐1‐1 TsukijiChuo‐kuTokyo104‐0045Japan; ^2^ Division of Hepatobiliary and Pancreatic OncologyNational Cancer Center Hospital5‐1‐1 TsukijiChuo‐kuTokyo104‐0045Japan

**Keywords:** Antitumor immunity, autologous hematopoietic stem cell transplantation, homeostatic proliferation, neutrophil, NK cell

## Abstract

Autologous hematopoietic stem cell transplantation (HSCT) can induce a strong antitumor immunity by homeostatic proliferation (HP) of T cells and suppression of regulatory T cells following preconditioning‐induced lymphopenia. However, the role of innate immunity including natural killer (NK) cells is still not understood. Here, first, we examined whether NK cells exert an antitumor effect after syngeneic HSCT in a murine colon cancer model. Flow cytometry showed that NK cells as well as T cells rapidly proliferated after HSCT, and the frequency of mature NK cells was increased in tumor during HP. Furthermore, NK cells undergoing HP were highly activated, which contributed to substantial tumor suppression. Then, we found that a large number of neutrophils accumulated in tumor early after syngeneic HSCT. It was recently reported that neutrophil‐derived mediators modulate NK cell effector functions, and so we examined whether the neutrophils infiltrated in tumor are associated with NK cell‐mediated antitumor effect. The depletion of neutrophils significantly impaired an activation of NK cells in tumor and increased the fraction of proliferative NK cells accompanied by a decrease in NK cell survival. The results suggested that neutrophils in tumor prevent NK cells from activation‐induced cell death during HP, thus leading to a significant antitumor effect by NK cells. This study revealed a novel aspect of antitumor immunity induced by HSCT and may contribute to the development of an effective therapeutic strategy for cancer using HSCT.

## Introduction

Autologous hematopoietic stem cell transplantation (HSCT) has been performed after high‐dose chemotherapy to rescue hematopoietic stem cells from myeloablative damage 1. Although it was generally assumed that the autologous nature of the graft precludes any immune‐mediated antitumor effect, graft‐versus‐host disease‐like reactions after autologous HSCT have been reported, suggesting that autologous HSCT induces autoimmunity 2, 3. In fact, several recent reports indicated that autologous HSCT can induce a strong antitumor immunity by homeostatic proliferation (HP) of T cells following preconditioning‐induced lymphopenia 4, 5. T‐cell HP is driven by the recognition of self‐antigens, and the availability of tumor antigens during HP leads to effective antitumor autoimmunity with specificity and memory 6, 7. The effect is mediated thorough an attenuation of the activation threshold of low‐affinity tumor‐specific T cells, allowing their preferential engagement and expansion 8.

Regarding other mechanisms of antitumor immunity induced by autologous HSCT, since lymphodepletion eradicates suppressive immune cells in the host including regulatory T cells (Tregs) and myeloid‐derived suppressor cells (MDSCs) 9, it is expected that a systemic reversal of immune tolerance contributes to the induction of antitumor immunity. Furthermore, we recently found that the frequency of CD4^+^ T cells expressing Foxp3 was significantly decreased in the tumor but not in the spleen after HSCT and that the decrease in Tregs was dependent on interleukin‐6 (IL‐6) produced by dendritic cells in the tumor. The results indicate that autologous HSCT creates an environment strongly supporting antitumor immunity in reconstituted lymphopenic recipients 10, 11. However, the mechanisms of antitumor immunity induced by the autologous HSCT are not fully understood. Especially, in addition to T cell‐mediated adaptive immunity, the role of innate immunity including natural killer (NK) cells is still unclear.

NK cells are a fundamental cellular component of innate immunity and are critical to cancer immunosurveillance and the clearance of virus‐infected cells. The function of NK cells is regulated by an array of activating receptors including NKG2D and inhibitory receptors including C‐type lectin NKG2A and the killer cell immunoglobulin‐like receptors (KIRs) 12. In the clinical setting of allogeneic HSCT, donor‐derived NK cells rapidly appeared during immune reconstitution after HSCT and exerted their graft‐versus‐tumor effects both by direct killing of tumor cells and by warning other cells via proinflammatory cytokines such as IFN‐*γ* and TNF‐*α* and cytokines such as MIP‐1*β* 13. It has been reported that in human leukocyte antigen (HLA)‐mismatched allogeneic HSCT, KIR ligand‐mismatched NK cells produced a strong antitumor effect 14. In autologous HSCT, it is unclear whether NK cells undergoing HP are actually associated with an induction antitumor effect 15.

In autologous HSCT, neutrophils are regarded as the first hematopoietic cells to reconstitute the immune system 16. Neutrophils are an important barrier in controlling infectious microorganisms such as bacteria and fungus by phagocytosis 17. In addition to the phagocytic activity, there has been increasing evidence that neutrophil‐derived mediators modulate NK cell effector functions 18, 19—neutrophils are required for homeostasis, maturation, and function of NK cells in human and mice 20. However, how the neutrophils contribute to the induction of antitumor immunity and how the neutrophils communicate with NK cells in the tumor microenvironment are not understood in autologous HSCT recipients.

In this study, we analyzed the characteristics of NK cells that infiltrated into tumor after HSCT and examined the role of NK cells on the antitumor effect of HSCT in a murine colon cancer xenograft model, especially focusing on the interaction between neutrophils and NK cells.

## Materials and Methods

### Animals and tumor cell lines

Seven‐ to 9‐week‐old female BALB/c mice were purchased from Charles River Japan (Kanagawa, Japan). Animal studies were carried out according to the Guideline for Animal Experiments of the National Cancer Center Research Institute and approved by the Institutional Committee for Ethics in Animal Experimentation. CT26 (American Type Culture Collection [ATCC], Rockville, MD) is a BALB/c‐derived colon cancer cell line and YAC‐1 (ATCC) is a mouse lymphoma cell line. They have been passaged and used in our laboratory for < 6 months after resuscitation, though these cell lines have not been authenticated by authors. Cells were maintained in RPMI 1640 medium containing heat‐inactivated 10% fetal bovine serum (FBS) (ICN Biomedicals, Irvine, CA), 2 mmol/L l‐glutamine, and 0.15% sodium bicarbonate (complete RPMI 1640).

### HSCT and tumor inoculation

Seven‐ to 9‐week‐old syngeneic mice received lethal (9 Gy) irradiation on the day of transplantation. The irradiated mice were injected intravenously with 5 × 10^6^ bone marrow (BM) cells and 4 × 10^6^ spleen cells from donor syngeneic mice. BM cells were isolated from donors by flushing each femur and tibia with RPMI 1640 medium supplemented with 10% FBS, and spleen cells were prepared by macerating the spleens. The transfer of splenocytes is crucial to induce antitumor immunity 4. CT26 cells (1 × 10^6^) were injected subcutaneously into the legs of the mice after HSCT. The tumor volume was calculated using the following formula: tumor volume = 1/2 × [(the shortest diameter)^2^ × (the longest diameter)]. Data are presented as mean ± standard deviation (SD).

### In vivo NK cell depletion

To deplete NK cells after syngeneic HSCT, the transplanted mice received intraperitoneal injections of 500 *μ*g of anti‐asialo GM1 antibody (Wako Pure Chemical Industries, Ltd., Osaka, Japan). Injections started on day 3 after syngeneic HSCT, and the treatment was repeated every 5 days throughout the entire experimental period to ensure depletion of NK cells. As shown by flow cytometry, ~100% of the NK cells were depleted in the spleen and tumor of the antibody‐treated mice (data not shown).

### Flow cytometry

For surface staining, anti‐mouse CD3e (clone 145‐2C11) conjugated with fluorescein isothiocyanate (FITC), phycoerythrin (PE), and PECy7; anti‐mouse CD3e (500A2) conjugated with Alexa Fluor 700; anti‐mouse CD11b (M1/70) conjugated with FITC and PE; anti‐mouse CD27 (LG.3A10) conjugated with PE‐Cy7; anti‐mouse CD45 (30‐F11) conjugated with Alexa Fluor 700; anti‐mouse CD49b (DX5) conjugated with FITC and PE; anti‐mouse NKG2A/C/E (20d5) conjugated with FITC; anti‐mouse CD314/NKG2D (CX5) conjugated with PE; anti‐mouse Ly6G (1A8) conjugated with FITC; and anti‐mouse CD107a (1D4B) conjugated with PE‐Cy7 were purchased from BD Biosciences (San Jose, CA). Tumors and spleens were harvested from the mice and prepared by mechanical dissociation. After washing, cells were incubated with monoclonal antibodies (mAbs) in a total volume of 100 *μ*L phosphate‐buffered saline (PBS) with 2% FBS for 30 min at 4°C. LIVE/DEAD Fixable Far Red Dead Cell Stain Kit (Invitrogen, Carlsbad, CA) was used for dead cell staining. Purified anti‐mouse CD16/CD32 (2.4G2) purchased from BD Biosciences was used for Fc receptor blocking before surface staining.

Intracellular molecules were stained using a BD Cytofix/Cytoperm Kit (BD Biosciences) with anti‐mouse IFN‐*γ* (XMG1.2) conjugated with PE (BD Biosciences) and anti‐mouse Ki‐67 (SolA15) conjugated with PE (eBioscience, San Diego, CA) according to the manufacturer's instructions. For the ex vivo NK cell restimulation assay, tumor‐infiltrating lymphocytes (TILs) were isolated by Histopaque (Sigma‐Aldrich, St. Louis, MO) gradient centrifugation of mechanically disaggregated tumor cells and cultured with YAC‐1 target cells (effector to target ratio, 10:1) at 37°C for 5 h in 96‐well plates in 200 *μ*L of complete RPMI 1640 medium in the presence of Brefeldin A (5 *μ*g/mL, Sigma‐Aldrich). Phorbol 12‐myristate 13‐acetate (100 ng/mL, Sigma‐Aldrich) and ionomycin (1.0 *μ*g/mL, Sigma‐Aldrich) were added for stimulation of cells as a positive control. Next, surface staining followed by IFN‐*γ* intracellular staining was performed. Flow cytometry was performed using an EC800 (Sony, Tokyo, Japan). FlowJo software (Tree Star Inc., Ashland, OR) was used for all flow cytometry analysis. Irrelevant IgG mAbs were used as a negative control.

### HE staining and immunohistochemistry

Tumors from mice were fixed in 10% neutral buffered formalin overnight and embedded in paraffin. Paraffin‐embedded blocks were cut into 5‐*μ*m‐thick sections and stained with hematoxylin and eosin (HE). Immunostaining was performed using streptavidin–biotin–peroxidase complex techniques (Nichirei, Tokyo, Japan). Consecutive cryostat tissue sections (6 *μ*m) were mounted on glass slides and fixed in 99.5% ethanol for 20 min. After blocking with normal rabbit serum, the sections were stained with anti‐mouse Gr‐1 antibody (BD Biosciences). The sections were counterstained with methyl green. Positive cells were counted in 10 representative high‐power fields (400×) under a microscope.

### In vivo neutrophil depletion

To deplete neutrophils after syngeneic HSCT, 150 *μ*g of anti‐Ly6G antibody (clone 1A8; Bio X cell, West Lebanon, NH) was injected intraperitoneally every other day from day 6 to day 12 after tumor inoculation. The dose of anti‐Ly6G antibody was determined by lethality in recipients of syngeneic HSCT using doses of 200 *μ*g or over, which are usually used for neutrophil depletion in steady‐state mice 21. As shown by flow cytometry, ~90% of the neutrophils were depleted in the antibody‐treated mice in the spleen and tumor (data not shown).

### Statistical analysis

Comparative analyses of the data were determined by the Student's *t*‐test, using SPSS statistical software (SPSS Japan Inc., Tokyo, Japan). *P *<* *0.05 was considered to be a significant difference.

## Results

### NK cells undergoing HP resulted in an antitumor effect after syngeneic HSCT

**Figure 1:**
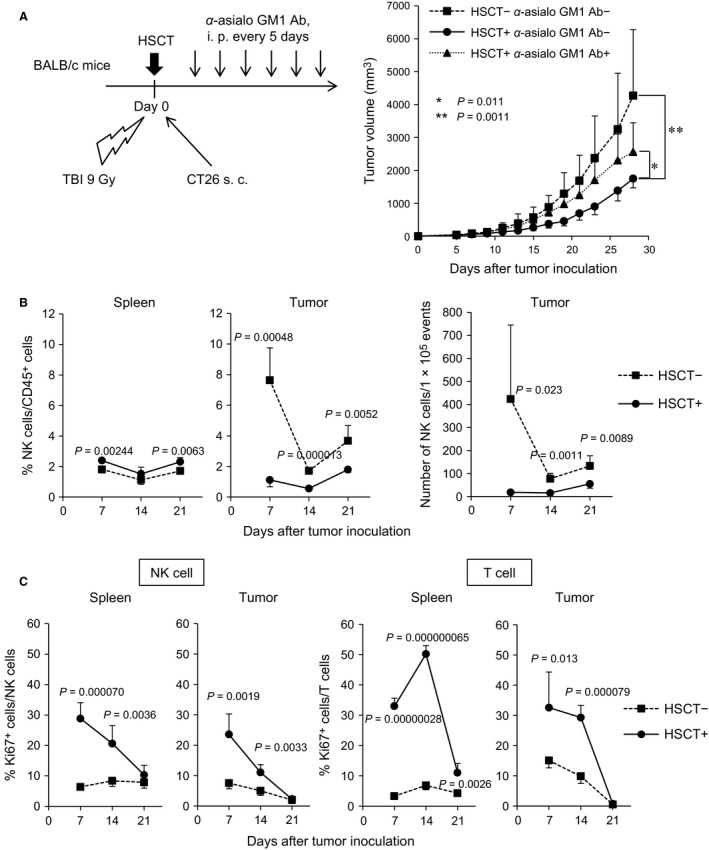
NK cells undergoing HP elicited a strong antitumor immunity after syngeneic HSCT. (A) Tumor growth suppression in syngeneic HSCT mice. BALB/c mice underwent syngeneic HSCT, followed by CT26 cell inoculation. The transplanted BALB/c mice were intraperitoneally injected with anti‐asialo GM1 antibody to deplete NK cells (number of animals per each group: *n* = 6–9). The tumor volume was measured at the indicated days after tumor inoculation. Data are shown as mean ± SD. (B) Frequency of NK cells in the spleen and tumor after syngeneic HSCT. The tumors and spleens at days 7, 14, and 21 after tumor inoculation were harvested and the frequency of NK cells was analyzed by flow cytometry (*n* = 4) (left panel). NK cells were defined as CD3^−^ DX5^+^ cells gated on live CD45^+^ cells. Number of NK cells within fixed number of tumor cells (1 × 10^5^) was measured by flow cytometry (right panel). (C) Ki67^+^ cells within NK cells and T cells in the spleen and tumor. Ki67 intracellular staining was performed and the frequency of Ki67^+^ NK cells and T cells was analyzed by flow cytometry (*n* = 4). T cells were defined as CD3^+^ DX5^−^ cells gated on live CD45^+^ cells. NK, natural killer; HP, homeostatic proliferation; HSCT, hematopoietic stem cell transplantation.

First, to examine the contribution of NK cells to antitumor effect after syngeneic HSCT, NK cells were depleted by intraperitoneal injection of anti‐asialo GM1 antibody in syngeneic HSCT recipients with murine colon cancer xenografts (Fig. 1A left). Tumor growth was significantly suppressed in the HSCT recipients as previously reported 10, 11. The tumor growth suppression was largely cancelled by the depletion of NK cells (Fig. 1A right), indicating that NK cells played an essential role in inducing an antitumor effect after HSCT.

We then examined the dynamics of NK cells in the spleen and tumor during 1–3 weeks after syngeneic HSCT. Flow cytometry showed that in the spleen the frequency of NK cells within live CD45^+^ hematopoietic cells was slightly increased as compared with the no‐treatment group (Fig. 1B left). In tumor, the frequency and number of NK cells within live CD45^+^ cells significantly decreased in HSCT recipients as compared with no‐treatment mice from early phase after HSCT (Fig. 1B middle and right). We examined the proliferative activity of NK cells and T cells, and flow cytometry showed that NK cells as well as T cells actively proliferated both in the spleen and in the tumor after HSCT (Fig. 1C and Fig. S1A–C), indicating that not only T cells but also NK cells undergo HP after syngeneic HSCT. The peak of Ki67^+^ frequency in NK cells was at day 7 after HSCT in the spleen and tumor, which was compatible with a previous report in which the peak of homeostatic expansion of NK cells was around 1 week after infusion of NK cells in a lymphopenic host 22. In T cells, the peak of Ki67^+^ frequency was at day 14, suggesting that NK cells began to proliferate earlier than did T cells.

### NK cells in HSCT tumor were highly activated

**Figure 2:**
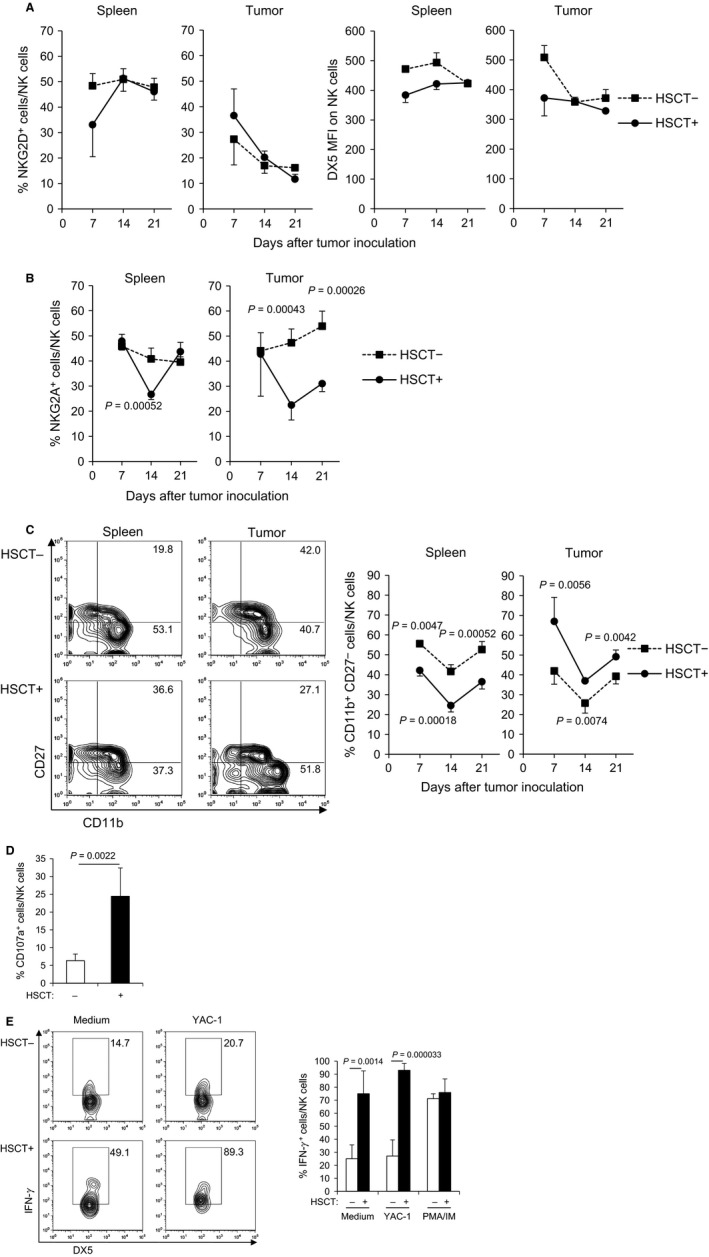
NK cells in HSCT tumor showed mature phenotype with a low expression level of inhibitory receptor and were highly activated. (A) The expression of activating receptors on NK cells in the spleen and tumor after syngeneic HSCT. The tumors and spleens at days 7, 14, and 21 after tumor inoculation were harvested and the frequency of NKG2D^+^ cells within NK cells and MFI of DX5 on NK cells was analyzed by flow cytometry (*n* = 4). (B) The expression of inhibitory receptor on NK cells in the spleen and tumor after syngeneic HSCT. The frequency of NKG2A^+^ cells within NK cells was analyzed by flow cytometry (*n* = 4). (C) Maturity of NK cells in the spleen and tumor after syngeneic HSCT. The frequency of mature NK cells (CD11b^+^ CD27^−^) within NK cells was analyzed by flow cytometry (*n* = 4). Representative flow cytometry plots of spleen and tumor at day 21 after tumor inoculation are shown (left panel). (D) Cytotoxicity of NK cells in tumor at day 14 after syngeneic HSCT. The percentage of CD107a^+^ cells within NK cells was analyzed by flow cytometry (*n* = 4). (E) Cytokine production of NK cells in tumor at day 14 after syngeneic HSCT. TILs isolated from tumors were restimulated with YAC‐1 tumor cells ex vivo. Then, IFN‐*γ* intracellular cytokine staining was performed and the frequency of IFN‐*γ* ^+^ NK cells was analyzed by flow cytometry (*n* = 4). Representative flow cytometry plots are shown (left panel). NK, natural killer; HSCT, hematopoietic stem cell transplantation; MFI, mean fluorescence intensity; TILs, tumor‐infiltrating lymphocytes.

Next, we assessed the expression of representative activating and inhibitory receptors on NK cells in the spleen and tumor. Flow cytometry showed that, in tumor, the frequency of activating receptor NKG2D^+^ NK cells and the expression level of DX5 on NK cells in HSCT recipients were similar to those in the no‐treatment group (Fig. 2A), whereas the frequency of inhibitory receptor NKG2A^+^ NK cells was markedly decreased in the tumor but not in the spleen of HSCT recipients (Fig. 2B). As NKG2A inhibits NK cell activation by binding to the major histocompatibility complex (MHC) class I 12, the decreased expression of NKG2A on NK cells suggested that NK cells in tumor of HSCT recipients (HSCT tumor) had a lower threshold for activation than cells in tumor of no‐treatment mice (non‐HSCT tumor) did, and led to an effective antitumor immunity in MHC class I^+^ tumors such as CT26.

We then analyzed the maturation status of NK cells after syngeneic HSCT, as their maturity is deeply related to the function of NK cells 23. Mature NK cells were defined as CD11b^+^ CD27^−^ cells 24. As DX5^−^ NK cells are reported to include CD11b^−^ immature NK cells and NK1.1 is not expressed in BALB/c strain mice 25, we assessed only the CD11b^+^ mature phenotype of NK cells. The frequency of CD11b^+^ CD27^−^ mature NK cells was significantly decreased in the spleen of syngeneic HSCT recipients compared with no‐treatment mice, whereas in contrast, it was increased in HSCT tumor (Fig. 2C). The increase of mature NK cells in tumor may be related to NK cell‐mediated antitumor immunity.

To further investigate whether these mature, proliferative NK cells in HSCT tumor are equipped with antitumor activity, the expression of cell surface marker CD107a, an indicator of cytotoxic activity, was examined on NK cells. Flow cytometry showed that the frequency of CD107a^+^ NK cells was significantly higher in HSCT tumor than in non‐HSCT tumor at day 14 (6.3% for non‐HSCT vs. 24.4% for HSCT; *P *=* *0.0022) (Fig. 2D). The TILs were isolated from tumors at day 14 after HSCT using density gradient centrifugation, and were restimulated with YAC‐1 target cells ex vivo. The frequency of IFN‐*γ* ^+^ cells within live NK cells in HSCT recipients was significantly higher as compared with that in no‐treatment mice, regardless of the YAC‐1 restimulation (27.1% for non‐HSCT vs. 93.0% for HSCT; *P *=* *0.000033) (Fig. 2E and Fig. S2), indicating that NK cells in HSCT tumor are endowed with cytotoxic and cytokine productive capacities. Although the number of NK cells accumulated into tumor sites was larger in non‐HSCT tumor than in HSCT tumor (Fig. 1B), the phenotypes of NK cells in non‐HSCT tumor were immature and hyporesponsive, suggesting that the NK cells in non‐HSCT tumor were in a state of dysfunction.

### A large number of neutrophils accumulated in HSCT tumor

**Figure 3:**
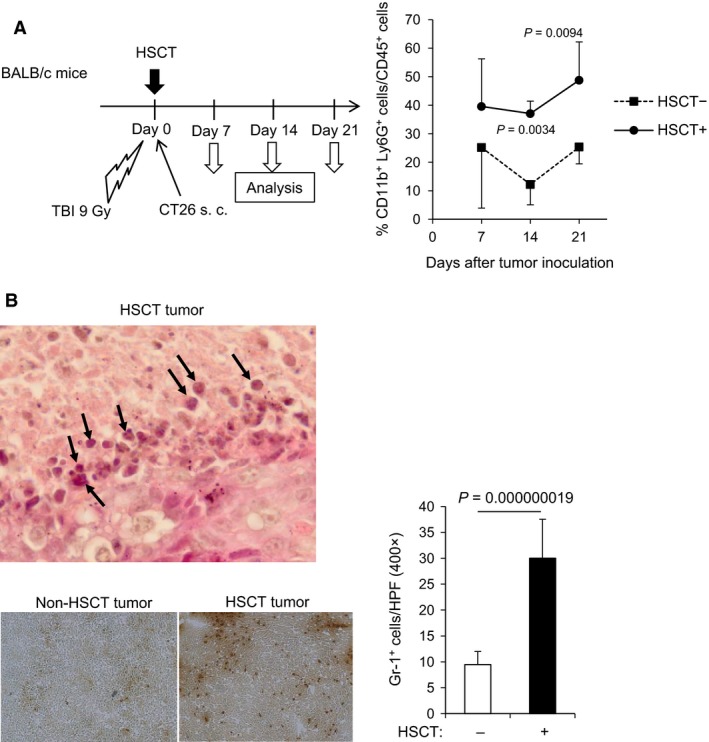
A large number of neutrophils accumulated in tumor early after syngeneic HSCT. (A) Frequency of CD11b^+^ Ly6G^+^ neutrophils within live CD45^+^ hematopoietic cells in tumor. HSCT tumors were harvested on days 7, 14, and 21, and mechanically dissociated to single‐cell suspension. The frequency of CD11b^+^ Ly6G^+^ cells within live CD45^+^ cells was assessed by flow cytometry (*n* = 4). (B) Neutrophil infiltration in HSCT tumor. Hematoxylin and eosin‐stained section of a representative tumor on day 14 after syngeneic HSCT is shown (upper panel). Arrows indicate neutrophils with lobulated nuclei. The frozen sections of tumor at day 14 after HSCT were processed for immunohistochemistry with anti‐mouse Gr‐1 antibody (lower left panel). Gr‐1^+^ cells were counted in 10 representative HPFs under a microscope (lower right panel). Original magnifications: 1000× (upper), 400× (lower left). HSCT, hematopoietic stem cell transplantation; HPF, high‐power field.

It has been recently reported that there is crosstalk between neutrophils and NK cells 20, and so we examined whether the neutrophils infiltrated in tumor are associated with NK cell‐mediated antitumor immunity. We first compared the frequency and number of neutrophils in HSCT tumor with those in non‐HSCT tumor. Flow cytometry revealed that the frequency of neutrophils (CD11b^+^ Ly6G^+^ cells) within live CD45^+^ hematopoietic cells was higher in HSCT tumor than in non‐HSCT tumor at days 14 and 21 (Fig. 3A). HE staining of sections of HSCT tumor showed that many neutrophils with lobulated nuclei infiltrated into tumor (Fig. 3B upper). Immunohistochemistry demonstrated that more Gr‐1^+^ cells were accumulated in HSCT tumor than in non‐HSCT tumor (Fig. 3B lower left), and the number of Gr‐1^+^ cells was significantly increased in HSCT tumor (Fig. 3B lower right). Although Gr‐1^+^ cells include neutrophils as well as small populations of Ly6C^+^ cells such as monocytic MDSCs and tumor‐associated macrophages 26, the frequency of CD11b^+^ Ly6C^+^ cells in HSCT tumor was similar to that in non‐HSCT tumor (data not shown). The results indicated that neutrophils preferentially accumulated in tumor from an early phase after syngeneic HSCT.

### Neutrophils enhanced NK cell activation and inhibited cell death after syngeneic HSCT

**Figure 4:**
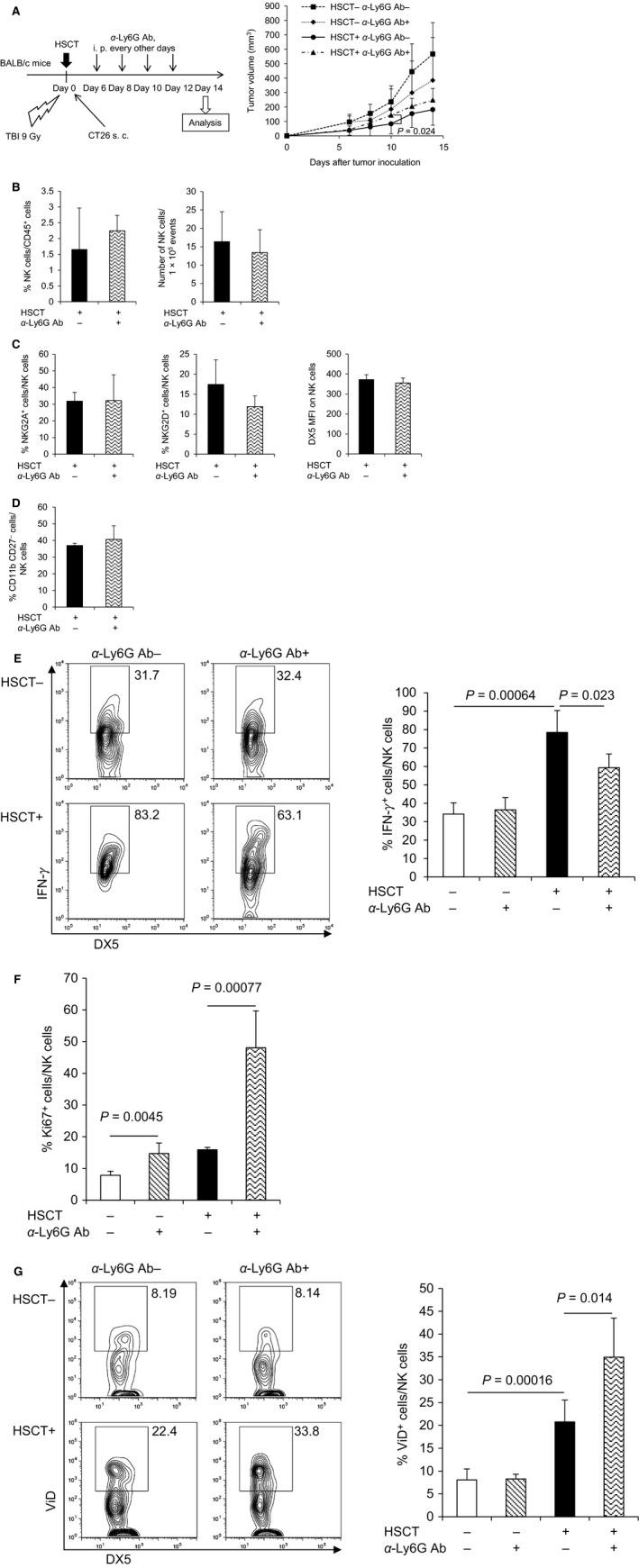
Neutrophil depletion attenuated activation of NK cells in HSCT tumor. Tumors were harvested at day 14 after syngeneic HSCT, and TILs were isolated from tumors and analyzed by flow cytometry. (A) Tumor growth suppression in the neutrophil‐depleted mice. Transplanted or non‐treated BALB/c mice were intraperitoneally injected with anti‐Ly6G antibody every other day after tumor inoculation to deplete neutrophils (number of animals per each group: *n* = 6–8). The tumor volume was measured at the indicated days after tumor inoculation. Data are shown as mean ± SD. (B) Frequency and number of NK cells in HSCT tumor after syngeneic HSCT with neutrophil depletion. The frequency of NK cells and number of NK cells within fixed number of tumor cells (1 × 10^5^) were analyzed (*n* = 4). (C) The expression of activating and inhibitory receptors on NK cells in HSCT tumor with neutrophil depletion. The frequency of NKG2A^+^ cells (left panel) and NKG2D^+^ cells (middle panel) within NK cells, and MFI of DX5 on NK cells (right panel) were analyzed (*n* = 4). (D) Maturity of NK cells in HSCT tumor with neutrophil depletion. The frequency of mature NK cells (CD11b^+^ CD27^−^) within NK cells was analyzed (*n* = 4). (E) IFN‐*γ* production of NK cells in HSCT tumor with neutrophil depletion. TILs isolated from tumors were restimulated with YAC‐1 tumor cells ex vivo. Then, IFN‐*γ* intracellular cytokine staining was performed and the frequency of IFN‐*γ* ^+^ NK cells was analyzed by flow cytometry (*n* = 3–4). Representative flow cytometry plots are shown (left panel). (F) Ki67^+^ cells within NK cells in HSCT tumor with neutrophil depletion. Ki67 intracellular staining was performed and the frequency of Ki67^+^ NK cells was analyzed (*n* = 4). (G) Dead cells within NK cells in HSCT tumor with neutrophil depletion. Dead cell staining was performed and the percentage of ViD^+^ dead NK cells was analyzed (*n* = 4). Representative flow cytometry plots are shown (left panel). NK, natural killer; HSCT, hematopoietic stem cell transplantation; TILs, tumor‐infiltrating lymphocytes; MFI, mean fluorescence intensity; ViD, viability dye.

To elucidate the effect of neutrophils on NK cell‐mediated antitumor immunity, neutrophils were depleted by an intraperitoneal injection of anti‐Ly6G antibody from days 6 to 12 after syngeneic HSCT (Fig. 4A left). The depletion of neutrophils increased tumor volumes in HSCT recipients, whereas the depletion tended to decrease tumor volumes in non‐HSCT mice, indicating that an in vivo role of neutrophils in tumor growth differed between HSCT recipients and non‐HSCT mice (Fig. 4A right). Flow cytometry showed that the depletion of neutrophils did not change the frequency or the number of NK cells within live CD45^+^ cells in tumor at day 14 (Fig. 4B). Furthermore, neither the frequency of NKG2A^+^ or NKG2D^+^ cells within live NK cells nor the expression of DX5 on NK cells was changed in HSCT tumor and non‐HSCT tumor (Fig. 4C). In addition, the maturity of NK cells in HSCT tumor was similar to that in non‐HSCT tumor (Fig. 4D).

To investigate the activation status of NK cells in neutrophil‐depleted HSCT recipients, TILs were isolated and restimulated with YAC‐1 cells ex vivo. Flow cytometry showed that the frequency of IFN‐*γ* ^+^ cells within NK cells in the tumor of neutrophil‐depleted HSCT recipients was significantly lower than non‐neutrophil‐depleted HSCT recipients (Fig. 4E and Fig. S3), suggesting that neutrophils were associated with NK cell activation after HSCT. Lastly, we examined the proliferative activity and cell death status of NK cells in tumor. Neutrophil depletion significantly increased the fraction of Ki67^+^ NK cells (Fig. 4F and Fig. S4) and also increased the fraction of dead NK cells at day 14 (Fig. 4G: 34.9% for neutrophil‐depleted HSCT vs. 20.8% for non‐neutrophil‐depleted HSCT; *P *=* *0.014). The results indicated that neutrophil depletion during HP induced proliferation and poor survival of NK cells. Intratumoral NK cells were more likely to die under HP induced by HSCT than the no‐treatment control, suggesting that HP‐induced proliferation is counterbalanced by cell death induction. Neutrophils in tumor may prevent NK cells from an activation‐induced cell death during HP, leading to the significant antitumor effect by NK cells.

## Discussion

In this study, we showed that NK cells undergoing HP were highly activated and exerted a substantial antitumor effect in syngeneic HSCT recipients. We found that a large number of neutrophils infiltrated into tumor early after HSCT. Neutrophils appeared to interact with NK cells in tumor and act as a brake on the induction of cell death of activated NK cells in HSCT recipients, potentially leading to the enhancement of antitumor effect. The results shed light on a novel role of innate immunity activation involved in the antitumor immunity after autologous HSCT.

Several mechanisms underlying the antitumor immunity induced by syngeneic HSCT have been reported 5. It has been reported that the immune reconstitution of autologous HSCT recipients is responsible for improved T‐cell priming and antitumor efficacy 4, 6, 7. In addition, we recently found that vascular endothelial growth factor (VEGF)‐D‐mediated suppression of Tregs in tumor is important for inducing an effective antitumor immunity after syngeneic HSCT 11. Except for these two T cell‐mediated mechanisms, the role of NK cells in inducing antitumor immunity has not been elucidated because T cells have been considered as the most important and potent immune cells involved in antitumor immunity. It is known that NK cells as well as neutrophils rapidly proliferate during the reconstitution of the immune system after autologous HSCT 16. Moreover, the actuation of innate immunity is generally faster than the effector phase of adaptive immunity, as the latter requires time for antigen‐priming. In fact, recovery of the number of NK cells in peripheral blood early after autologous HSCT was reported to be the prognostic factor among patients with non‐Hodgkin lymphoma in a prospective study 27. The activation of NK cells probably leads to significant tumor growth suppression in the early phase after HSCT, while T cell‐mediated acquired immunity further enhances the antitumor effect in a later phase.

In this study, NK cells began to proliferate in the early phase and the proliferation continued within 3 weeks after HSCT (Fig. 1C), while the frequency of CD107a^+^ NK cells was significantly higher in HSCT tumor than in non‐HSCT tumor at day 14 (Fig. 2D) but not at day 21 (data not shown). The results indicated that NK cells in tumor were highly activated during HP, which is in line with a report that homeostatically proliferating NK cells showed high cytotoxic activity and produced IFN‐*γ* without receptor triggering in a murine lymphopenia model, suggesting that the proliferative forces alone are able to activate NK cells 22. In addition to the enhanced proliferation, NK cells in HSCT tumor were found to be a mature phenotype with a low expression level of inhibitory receptor NKG2A (Fig. 2B and C). It was reported that NKG2A was upregulated on NK cells in peripheral blood early after haplo‐identical allogeneic HSCT, which was associated with immaturity and poor alloreactivity 28, 29. The population of proliferating NK cells with a mature phenotype and low expression level of inhibitory receptors may lead to an effective antitumor immunity in HSCT tumor.

Gill et al. reported that the adaptive transfer of murine NK cells alone failed to control tumor growth in HSCT, and that this NK cell dysfunction was related to loss of cytotoxicity, which progressed with tumor exposure 30. In this study, NK cells showed enhanced cytotoxicity and IFN‐*γ* production after syngeneic HSCT (Fig. 2D and E), while the depletion of neutrophils did not change the maturity or receptor expression of NK cells; however, it induced NK cell proliferation accompanied by a decrease in NK cell survival and suppressed cytokine production during HP. Therefore, marked accumulation of neutrophils in tumor may play an important role in preventing NK cells from dysfunction during HP. Several cytokines from neutrophils are reported to activate NK cells or support survival of NK cells. IL‐15 is regarded as a most important cytokine for maintenance of NK cells 31, and IL‐18 activates NK cells in synergy with IL‐12 produced from dendritic cells 32. In addition, we showed in a previous study that a proinflammatory cytokine S100A8/A9, which is constitutively expressed by myeloid cells including neutrophils, can enhance activation of NK cells via interaction with a receptor for advanced glycation end products (RAGE) 33. As our next step, we are planning to clarify the molecular mechanism of NK cell activation and survival supported by neutrophils after HSCT, focusing on the combinational effect of such cytokines.

In conclusion, in the early period after syngeneic HSCT, NK cells play a major role in the antitumor effect. The neutrophils in tumor may support the sustained antitumor effect of NK cells. This novel relationship reveals an important aspect of antitumor immunity induction in HSCT recipients and may contribute to the development of effective therapeutic strategies for cancer using HSCT.

## Conflict of Interest

None declared.

## Supporting information


**Figure S1.** Ki67^+^ cells within NK cells and T cells in the spleen and tumor. The frequency of Ki67^+^ NK cells and T cells was analyzed by flow cytometry. As the cut‐off line of Ki67^+^ cells is unclear, representative dot plots of isotype control and Ki67^+^ cells in the spleen and tumor at days 7 (A), 14 (B), and 21 (C) after tumor inoculation are shown. NK cells were defined as CD3^−^ DX5^+^ cells gated on live CD45^+^ cells. T cells were defined as CD3^+^ DX5^−^ cells gated on live CD45^+^ cells.
**Figure S2.** IFN‐γ production of NK cells in tumor. TILs isolated from tumors at day 14 after syngeneic HSCT were restimulated with YAC‐1 cells ex vivo. The frequency of IFN‐γ^+^ NK cells was analyzed by flow cytometry. As the cut‐off line of IFN‐γ^+^ cells is unclear, a representative dot plot of isotype control of splenocytes is shown.
**Figure S3.** IFN‐γ production of NK cells in tumor with neutrophil depletion. TILs isolated from tumors at day 14 after syngeneic HSCT were restimulated with YAC‐1 cells ex vivo. The frequency of IFN‐γ^+^ NK cells was analyzed by flow cytometry. A representative dot plot of isotype control of splenocytes is shown.
**Figure S4.** Ki67^+^ cells within NK cells in tumor with neutrophil depletion at day 14 after syngeneic HSCT. The frequency of Ki67^+^ NK cells was analyzed. Representative dot plots of isotype control and Ki67^+^ cells in tumor are shown.
