# Supplementary material for: Interaction of natural killer cells with neutrophils exerts a significant antitumor immunity in hematopoietic stem cell transplantation recipients
Source: Cancer Med. 2015 Nov 21;5(1):49–60. doi: 10.1002/cam4.550 (PMC4708905; doi:10.1002/cam4.550)
Supplement: Supplementary file 1 — Figure S1. Ki67+ cells within NK cells and T cells in the spleen and tumor. The frequency of Ki67+ NK cells and T cells was analyzed by flow cytometry. As the cut‐off line of Ki67+ cells is unclear, representative dot plots of isotype control and Ki67+ cells in the spleen and tumor at days 7 (A), 14 (B), and 21 (C) after tumor inoculation are shown. NK cells were defined as CD3− DX5+ cells gated on live CD45+ cells. T cells were defined as CD3+ DX5− cells gated on live CD45+ cells. Figure S2. IFN‐γ production of NK cells in tumor. TILs isolated from tumors at day 14 after syngeneic HSCT were restimulated with YAC‐1 cells ex vivo. The frequency of IFN‐γ+ NK cells was analyzed by flow cytometry. As the cut‐off line of IFN‐γ+ cells is unclear, a representative dot plot of isotype control of splenocytes is shown. Figure S3. IFN‐γ production of NK cells in tumor with neutrophil depletion. TILs isolated from tumors at day 14 after syngeneic HSCT were restimulated with YAC‐1 cells ex vivo. The frequency of IFN‐γ+ NK cells was analyzed by flow cytometry. A representative dot plot of isotype control of splenocytes is shown. Figure S4. Ki67+ cells within NK cells in tumor with neutrophil depletion at day 14 after syngeneic HSCT. The frequency of Ki67+ NK cells was analyzed. Representative dot plots of isotype control and Ki67+ cells in tumor are shown. [file CAM4-5-049-s001.pdf]

Supplemental Figure S1

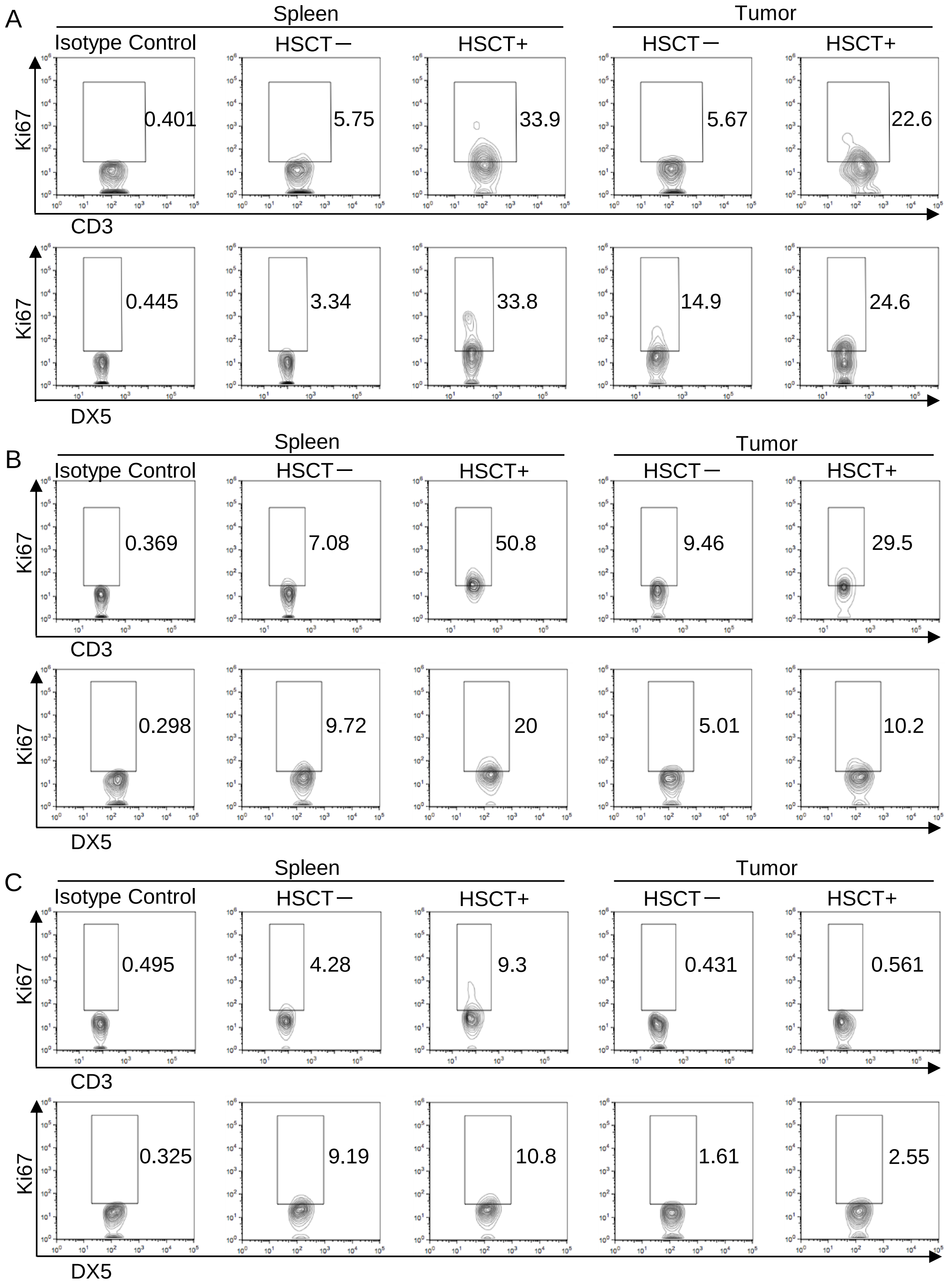


**Supplemental Figure S1.** Ki67$^+$ cells within NK cells and T cells in the spleen and tumor. The frequency of Ki67$^+$ NK cells and T cells was analyzed by flow cytometry. Since the cut-off line of Ki67$^+$ cells is unclear, representative dot plots of isotype control and Ki67$^+$ cells in the spleen and tumor at days 7 (A), 14 (B), and 21 (C) after tumor inoculation are shown. NK cells were defined as CD3$^-$ DX5$^+$ cells gated on live CD45$^+$ cells. T cells were defined as CD3$^+$ DX5$^-$ cells gated on live CD45$^+$ cells.

Supplemental Figure S2

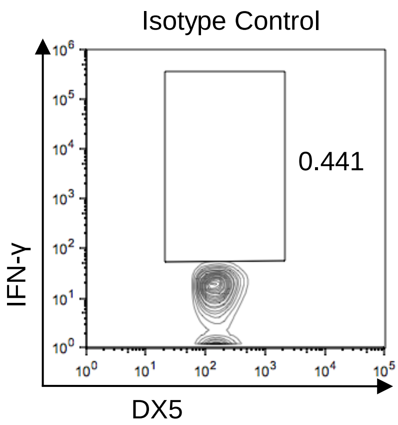


**Supplemental Figure S2.** IFN-γ production of NK cells in tumor. TILs isolated from tumors at day14 after syngeneic HSCT were restimulated with YAC-1 cells *ex vivo*. The frequency of IFN-$\gamma^+$ NK cells was analyzed by flow cytometry. Since the cut-off line of IFN-$\gamma^+$ cells is unclear, a representative dot plot of isotype control of splenocytes is shown.

Supplemental Figure S3

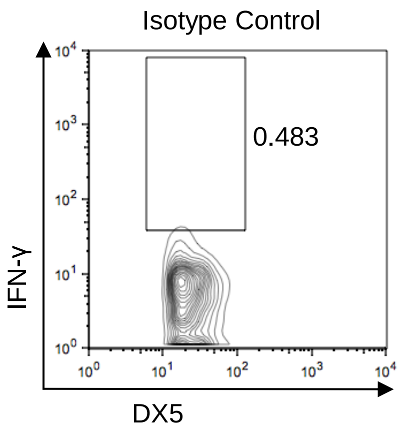


**Supplemental Figure S3.** IFN-γ production of NK cells in tumor with neutrophil depletion. TILs isolated from tumors at day14 after syngeneic HSCT were restimulated with YAC-1 cells *ex vivo*. The frequency of IFN-$\gamma^+$ NK cells was analyzed by flow cytometry. A representative dot plot of isotype control of splenocytes is shown.

Supplemental Figure S4

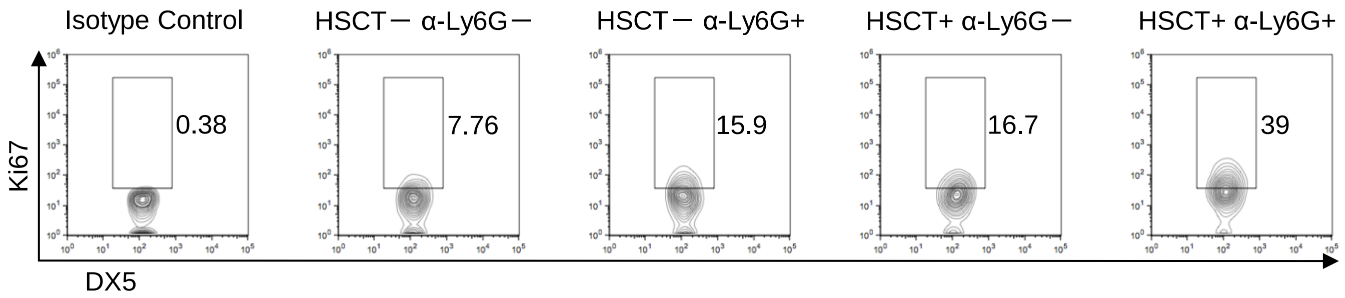


**Supplemental Figure S4.** Ki67$^{+}$ cells within NK cells in tumor with neutrophil depletion at day14 after syngeneic HSCT. The frequency of Ki67$^{+}$ NK cells was analyzed. Representative dot plots of isotype control and Ki67$^{+}$ cells in a tumor are shown.
